# Circumferential Thoracic Cord Compression Due to Combined Ossified Yellow Ligament and Ossified Posterior Longitudinal Ligament Managed by a Posterior-Only Approach

**DOI:** 10.7759/cureus.102360

**Published:** 2026-01-26

**Authors:** Sargunan B, Vishnu Prasath, Thomas John

**Affiliations:** 1 Spine Surgery, SKS Hospital and Postgraduate Medical Institute, Salem, IND

**Keywords:** ossified posterior longitudinal ligament, ossified yellow ligament, posterior only approach, surgical case reports, thoracic cord compression

## Abstract

Thoracic myelopathy caused by combined ossified yellow ligament (OYL) and ossified posterior longitudinal ligament (OPLL) is exceptionally rare and presents unique diagnostic and surgical challenges. We report a 45-year-old female with rapidly progressive gait disturbance and severe myelopathy found to have circumferential spinal cord compression at T3-T4 due to simultaneous dorsal OYL and ventral OPLL. MRI and CT imaging were essential in defining the extent of compression and ossification. Considering the morbidity of anterior thoracic exposure, a tailored posterior-only approach incorporating laminectomy, OYL excision, costotransversectomy-assisted partial OPLL decompression, and instrumented stabilization was performed, achieving adequate circumferential release. The patient demonstrated meaningful neurological recovery, improving from Nurick Grade 5 to Grade 3 within three months. This case highlights the importance of early recognition, comprehensive imaging, and individualized posterior-based strategies in managing complex upper thoracic stenosis caused by dual ossified pathologies.

## Introduction

Ossified yellow ligament (OYL) and ossified posterior longitudinal ligament (OPLL) are uncommon but well-recognized causes of thoracic myelopathy, with the thoracic spine being a relatively rare site compared to cervical involvement [1]. When both pathologies occur simultaneously, they can create circumferential spinal cord compression, leading to rapidly progressive neurological compromise. The narrow thoracic canal and limited vascular supply further predispose patients to severe deficits even with modest compression [2]. Although anterior or combined surgical approaches have traditionally been recommended for ventral lesions, they carry significant morbidity in the upper thoracic region due to complex anatomy. Emerging evidence supports posterior-only strategies, such as laminectomy, costotransversectomy, and targeted excision of ossified structures, as effective options capable of achieving adequate decompression in selected cases [3]. Reporting rare presentations like concurrent OYL and OPLL enhances clinical recognition and guides more tailored surgical decision-making.

## Case presentation

A 45-year-old female presented with a three-month history of progressive gait disturbance, which had advanced to the point where she had become bedridden for two months prior to evaluation. She reported worsening imbalance, stiffness in both lower limbs, and increasing difficulty initiating steps. She denied bowel or bladder incontinence, but described a sensation of heaviness in both legs, with intermittent mid-thoracic discomfort. Her symptoms had no identifiable inciting trauma. On neurological examination, the patient was alert and oriented. She demonstrated spastic paraparesis with Medical Research Council (MRC) grade 2/5 strength in hip flexors and knee extensors bilaterally, and 1/5 in ankle dorsiflexion. Tone was markedly increased, and bilateral Babinski signs were present. Deep tendon reflexes were brisk in the lower limbs, with sustained clonus at both ankles. Sensory testing revealed impaired proprioception and vibration in both feet, with a sensory level around T4. Her gait could not be tested as she was non-ambulatory. She was classified as Nurick Grade 5, consistent with severe myelopathy. The patient did not report neck pain, upper-extremity radicular pain, hand clumsiness, or dermatomal sensory symptoms suggestive of cervical radiculopathy or dominant cervical myelopathy. There was no history of bowel or bladder incontinence or urinary retention at presentation.

Baseline laboratory investigations, including complete blood count, electrolytes, renal and liver function tests, were within normal limits. Inflammatory markers were not elevated, reducing suspicion for infectious or inflammatory causes of myelopathy. Coagulation profile was normal, allowing safe operative planning (Table 1).

**Table 1:** Laboratory value of the patient. WBC: white blood cell; MCV: mean corpuscular volume; AST: aspartate aminotransferase; ALT: alanine aminotransferase; ALP: alkaline phosphatase; CRP: C-reactive protein; PT: prothrombin time; INR: international normalized ratio; aPTT: activated partial thromboplastin time; g/dL: grams per deciliter; mg/dL: milligrams per deciliter; U/L: units per liter; mmol/L: millimol per liter; µL: microliter

Parameter	Value	Reference Range
Hemoglobin	13.5	13-17 g/dL (M)/12-15 g/dL (F)
Total WBC Count	7500	4,000-11,000/µL
Platelets	253000	150,000-450,000/µL
Hematocrit	42%	40-50% (M)/36-44% (F)
MCV	88 fL	80-100 fL
Urea	30 mg/dL	10-40 mg/dL
Creatinine	0.9 mg/dL	0.6-1.2 mg/dL
Sodium	140 mmol/L	135-145 mmol/L
Potassium	4.2 mmol/L	3.5-5.0 mmol/L
Total Bilirubin	0.8 mg/dL	0.3-1.2 mg/dL
AST	30 U/L	5-40 U/L
ALT	28 U/L	5-40 U/L
ALP	90 U/L	44-147 U/L
Albumin	4.2 g/dL	3.5-5.2 g/dL
CRP	2 mg/dL	<5 mg/L
Calcium	9.5 mg/dL	8.6-10.2 mg/dL
Magnesium	2.0 mg/dL	1.7-2.4 mg/dL
PT	12 sec	11-13.5 sec
INR	1.0 sec	0.8-1.2
aPTT	30 sec	25-35 sec

**Figure 1:**
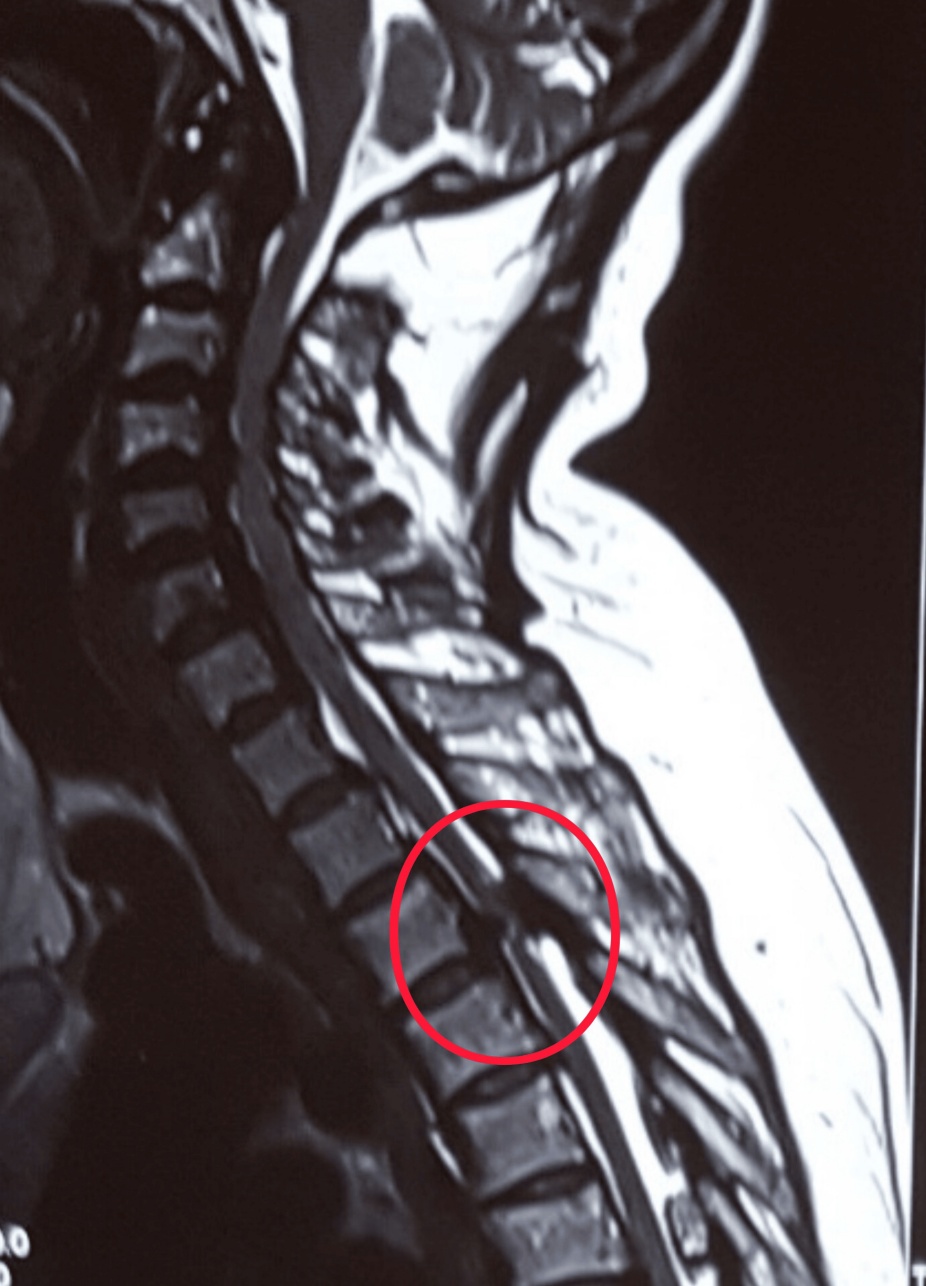
Pre-operative magnetic resonance imaging (MRI) demonstrates severe spinal cord compression at the T3-T4 level with associated T2 signal change (red circle). T3 denotes thoracic vertebral segment 3, and T4 denotes thoracic vertebral segment 4.

**Figure 2:**
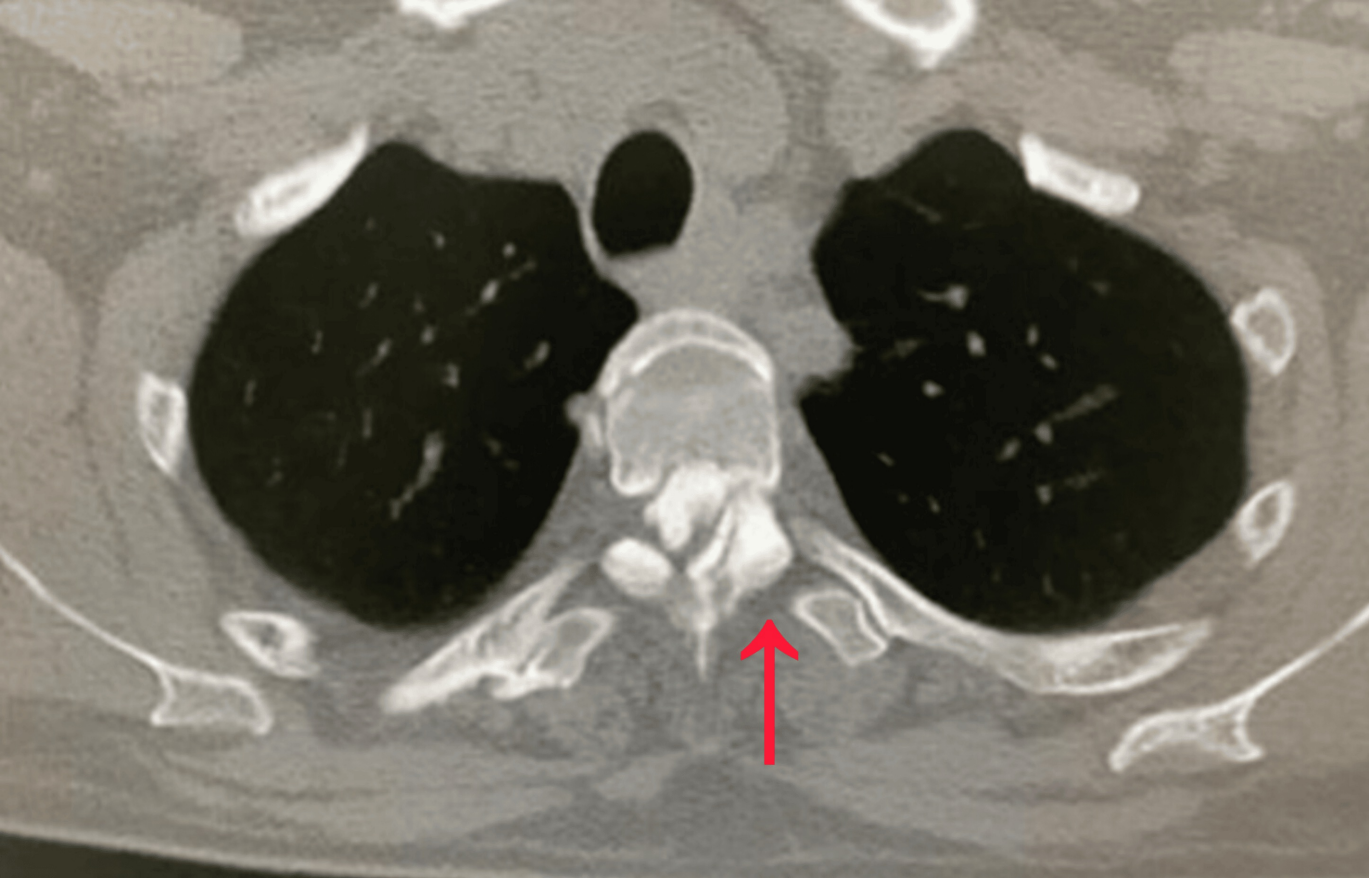
Pre-operative computed tomography (CT) demonstrates circumferential spinal canal compromise at the T3-T4 level due to anterior OPLL and posterior OYL (red arrow). OPLL: ossified posterior longitudinal ligament; OYL: ossified yellow ligament

MRI of the thoracic spine demonstrated severe circumferential spinal cord compression at the T3-T4 level, with both anterior and posterior elements contributing (Figure 1). Posterior compression corresponded to OYL, while CT imaging confirmed the presence of OPLL anteriorly (Figure 2). Together, these produced a tight canal resulting in marked cord flattening and a high T2 cord signal suggestive of myelopathy. The combination of OYL and OPLL at the upper thoracic level made this a rare presentation and posed significant surgical planning challenges.

Given the morbidity associated with anterior thoracic exposure, a posterior-only surgical approach was selected, aiming for both dorsal decompression and access for limited ventral decompression through a costotransversectomy window. Under general anesthesia, the patient was placed prone. A midline incision was made, and subperiosteal dissection exposed the T2-T5 posterior elements. Pedicle screws were inserted bilaterally at T2, T3, T4, and T5 for stabilization. Although imaging demonstrated extensive continuous cervical ossification, the absence of concordant upper-limb neurological deficits and the presence of severe thoracic canal compromise with cord signal changes supported thoracic myelopathy as the primary contributor to the patient’s rapid neurological decline, and thoracic decompression was therefore prioritized.

**Figure 3:**
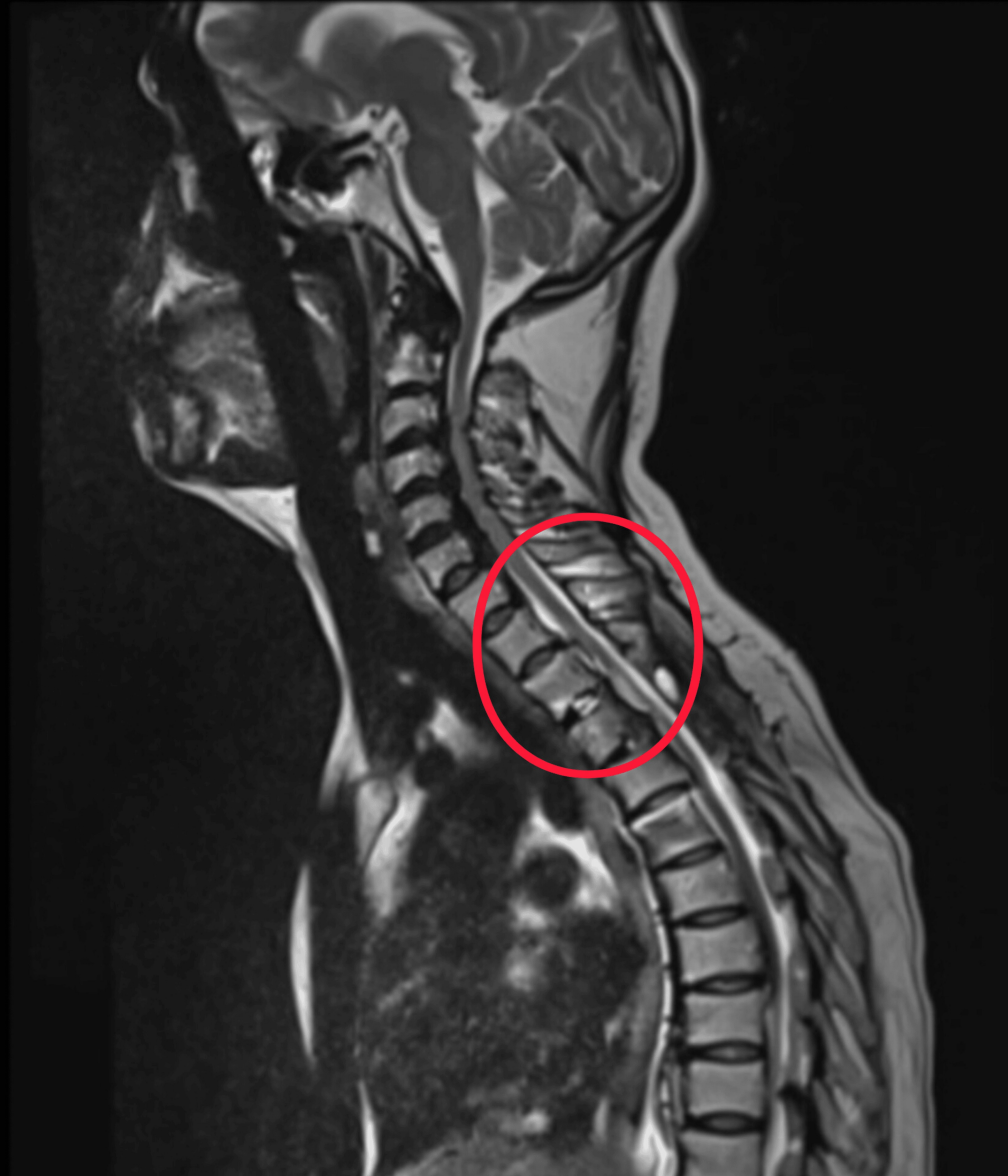
Post-operative magnetic resonance imaging (MRI) demonstrates restoration of CSF space and relieved spinal cord compression at the T3-T4 level (red circle). CSF: cerebrospinal fluid

**Figure 4:**
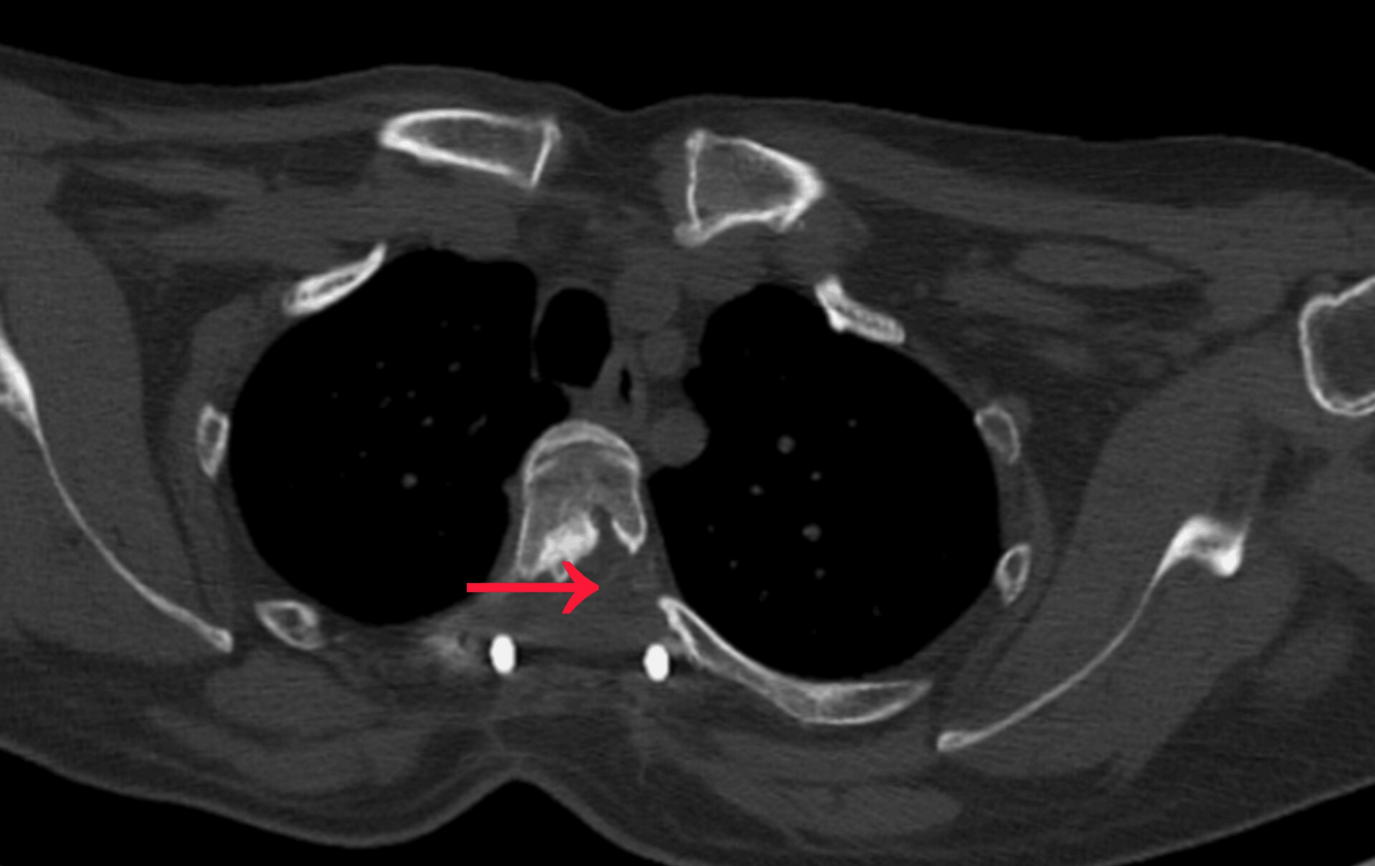
Post-operative computed tomography (CT) confirms adequate decompression (red arrow).

A laminectomy of T3 and T4 was performed, followed by careful removal of the OYL, achieving immediate posterior decompression. To address the anterior OPLL, a left-sided costotransversectomy was performed at T3-T4. The rib head and transverse process were resected, creating a corridor to the ventral canal. Using high-speed burr and microdissectors, a partial debulking of the OPLL was safely achieved without durotomy. Decompression at the T3-T4 level was performed in a controlled manner, recognizing the vulnerability of the thoracic spinal cord and the limited tolerance for manipulation at this level. Adequate circumferential decompression of the cord was confirmed. Rods were secured, and the wound closed in layers. The surgery achieved stable fixation and satisfactory canal expansion as confirmed on postoperative imaging (Figures 3-4).

The patient had an uneventful recovery and was mobilized with physiotherapy support. There was no new neurological deficit. Over three months, she demonstrated significant improvement in lower limb strength and gait function. At follow-up, she had improved to Nurick Grade 3, becoming ambulatory with walker assistance, reflecting meaningful neurological recovery attributable to timely decompression and stabilization. While this case demonstrates favorable neurological recovery following timely posterior decompression, conclusions should be interpreted cautiously, as outcomes may differ in patients with multilevel disease or concurrent cervical myelopathy.

## Discussion

Thoracic myelopathy remains a challenging clinical entity due to the complex anatomical structure of the thoracic spine, its relatively narrow canal, and the limited vascular reserve of the thoracic spinal cord. These anatomical features make the cord particularly susceptible to compressive injury, even when the degree of stenosis is modest [4]. The present case is unique because circumferential compression at the T3-T4 level was produced by two distinct ossified pathologies simultaneously, OYL dorsally and OPLL ventrally. Each of these conditions individually is an established cause of thoracic myelopathy, but their coexistence at the same level is exceedingly rare [5]. This combined pathology results in near-complete canal compromise and leaves minimal space for neural accommodation, explaining the rapid deterioration from gait imbalance to complete non-ambulation.

OYL typically arises from chronic hypertrophy and calcification of the ligamentum flavum, progressing in some patients to true ossification via endochondral pathways. OPLL, on the other hand, involves progressive pathological ossification of the posterior longitudinal ligament, frequently influenced by mechanical stress, metabolic imbalance, and genetic predisposition [6]. When both processes occur concurrently, the resulting circumferential stenosis leads to a more aggressive clinical course than seen with either pathology alone. In this case, the patient’s profound spastic paraparesis, sensory level, and brisk reflexes reflect the hallmark features of long-tract involvement in thoracic cord compression.

Radiological evaluation is fundamental in distinguishing between soft tissue hypertrophy and true ossification. MRI remains the preferred modality for detecting cord signal changes and assessing the severity of compression, while CT imaging provides superior characterization of ossified structures [7]. CT was crucial in this case for confirming the dual pathology and planning an operative approach that would allow safe access to both anterior and posterior compressive elements. Reliance on MRI alone may underestimate the extent of ossification, leading to incomplete decompression.

The choice of surgical approach in thoracic OPLL remains controversial, particularly at upper thoracic levels where anterior exposure is technically demanding. Traditional anterior decompression for ventral pathology provides direct access but carries significant morbidity due to the need for thoracotomy, manubrial elevation, or complex transthoracic techniques [8]. A study showed that complications, including respiratory distress, bleeding, and postoperative pain, are more common with anterior approaches in this region [8]. For this reason, posterior-only strategies have gained acceptance when carefully planned and executed.

Compared with anterior or combined approaches described in the literature, a posterior-only decompression avoids thoracotomy-related morbidity but requires advanced surgical expertise due to limited visualization and higher technical complexity when addressing circumferential compression.

In the present case, a posterior-only approach combining laminectomy, OYL excision, and costotransversectomy provided a balanced solution. Laminectomy allowed immediate dorsal decompression, while costotransversectomy created a lateral corridor through which partial OPLL debulking could be performed. This technique avoids the morbidity of anterior access while still addressing ventral compression adequately. A study demonstrated that costotransversectomy-assisted decompression can achieve excellent neurological outcomes in selected thoracic OPLL cases [9]. Instrumentation from T2 to T5 was essential to maintain stability following bone removal and to prevent postoperative kyphotic deformity, which can worsen cord stretch and compromise long-term outcomes [10].

Surgical management of circumferential thoracic cord compression due to combined OYL and OPLL is technically demanding, with risks including dural tears, cerebrospinal fluid leakage, neurological worsening, and incomplete decompression. Careful preoperative planning, gentle handling of the spinal cord, and awareness of potential dural adhesions are essential risk-mitigation strategies when operating at the upper thoracic level [10].

A key surgical principle illustrated by this case is the shift toward “functional decompression” rather than complete resection of OPLL. Full removal often risks dural tear or cord manipulation, particularly when ossification adheres to the dura. Partial thinning or “floating” of the OPLL, combined with posterior decompression, often produces sufficient canal expansion to relieve symptoms [11]. This strategy is especially relevant in upper thoracic levels, where full ventral resection carries a significantly higher risk.

Postoperative neurological improvement in this patient from Nurick Grade 5 to Grade 3 within three months highlights the potential for meaningful recovery even in severe thoracic myelopathy when decompression is timely. Although prolonged myelopathy frequently leads to irreversible damage, studies have shown that early surgical intervention, even in advanced cases, improves functional outcomes by halting ongoing cord injury and allowing residual neural pathways to recover [4].

This report is limited by its single-case design, short follow-up duration, and lack of long-term functional outcome measures, which restricts the broad generalization of the findings to all patients with complex thoracic myelopathy.

This case differs from typical thoracic myelopathy presentations due to the simultaneous presence of two ossified pathologies causing complete circumferential compression at an upper thoracic level. Moreover, the successful management using a single-stage posterior-only approach underscores the importance of individualized surgical planning. It demonstrates that even rare and complex patterns of thoracic stenosis can be managed effectively without necessitating anterior exposure when anatomical corridors are used judiciously.

## Conclusions

This case highlights the diagnostic and surgical challenges associated with rare circumferential thoracic cord compression caused by concurrent OYL and OPLL. The upper thoracic spine, with its narrow canal and limited vascular supply, is particularly vulnerable to rapid neurological deterioration once compressive pathology develops. Early recognition of progressive gait disturbance, spastic paraparesis, and a clear sensory level is essential for timely diagnosis. Comprehensive imaging with both MRI and CT is crucial for defining the extent of ossification and planning an effective surgical strategy. In this case, a single-stage posterior-only approach incorporating laminectomy, OYL excision, costotransversectomy, and stabilization allowed adequate circumferential decompression without the morbidity associated with anterior thoracic exposure. The patient’s significant functional recovery demonstrates that meaningful neurological improvement is achievable even in severe myelopathy when decompression is performed promptly. This case reinforces the importance of individualized, anatomy-guided surgical decision-making in managing complex thoracic spinal pathologies.
